# Comparative metabolomics and transcriptomics provide new insights into florpyrauxifen-benzyl resistance in *Echinochloa glabrescens*

**DOI:** 10.3389/fpls.2024.1392460

**Published:** 2024-07-03

**Authors:** Wenyong Jin, Kexin Xie, Wei Tang, Yongjie Yang, Jianping Zhang, Xiaoyue Yu, Yongliang Lu

**Affiliations:** State Key Laboratory of Rice Biology and Breeding, China National Rice Research Institute, Hangzhou, China

**Keywords:** *Echinochloa glabrescens*, florpyrauxifen-benzyl, metabolomics, transcriptomics, resistance, barnyard grass, herbicide

## Abstract

*Echinochloa glabrescens* Munro ex Hook. f. is a weed of the genus *Echinocloa* (*Echinocloa* spp.) that occurs frequently in paddy fields, causing serious harm to rice production. Florpyrauxifen-benzyl (FPB) is a foliar-applied herbicide used to control *Echinocloa* spp. in paddy fields. However, in recent years, with the widespread use of FPB in rice production, FPB-resistant barnyard grasses have been reported. Here, we identified an FPB-resistant *E. glabrescens* population with a resistance index (RI) of 10.65 and conducted a comparative analysis using untargeted metabolomics and transcriptomics to investigate the differences between an FPB-resistant *E. glabrescens* population and a susceptible *E. glabrescens* population after treatment with the recommended field dose of FPB. Our results showed that the FPB-resistant *E. glabrescens* had 115 differentially accumulated metabolites (DAMs; 65 up-regulated and 50 down-regulated) and 6397 differentially expressed genes (DEGs; 65 up-regulated and 50 down-regulated) compared to the susceptible *E. glabrescens.* The analysis of DAMs and DEGs revealed that DAMs were significantly enriched in Glutathione metabolism, Arginine and proline metabolism, and Zeatin biosynthesis pathways, while DEGs were mainly enriched in carbon fixation in photosynthetic organisms, photosynthesis, cyanoamino acid metabolism and glutathione metabolism, etc. The glutathione metabolism pathway was found to be significantly enriched for both DEGs and DAMs. Within this pathway, the metabolites (spermine) and genes (GSTU8, GSTU18, GSTF1) may play a pivotal role in the resistance mechanism of FPB-resistant *E. glabrescens*. Furthermore, we demonstrated the presence of GST-mediated metabolic resistance in an FPB-resistant *E. glabrescens* population by using NBD-Cl. Overall, our study provides new insights into the underlying mechanisms of *E. glabrescens* resistance to FPB through a comparative analysis of untargeted metabolomics and transcriptomics. Additionally, we identified the GST-mediated metabolic resistance in an FPB-resistant *E. glabrescens* population, and screened for three candidate genes (GSTU8, GSTU18, GSTF1), which has significant implications for improving the weed management efficacy of FPB in rice production and guiding judicious herbicide usage.

## 1 Introduction

Rice is one of the most important staple food crops globally and is the primary food for people in southern and northeastern China (National Bureau of Statistics, 2022). Barnyard grass (*Echinocloa* spp.) is the weed that is most detrimental to paddy fields, significantly restricting rice yield and quality (Michael, 1983; Lim et al., 2021). *Echinochloa glabrescens* Munro ex Hook.f., an annual species of Poaceae, is a common barnyard grass found in rice fields, commonly known as *Echinochloa crus-galli* var. *Formosensis* (Li et al., 2023). Based on a survey and research conducted by Chinese scholars from 2015 to 2018 that investigated the distribution characteristics of barnyard grass in rice fields across nine provincial administrative regions in China, 525 rice fields at 73 locations were examined (Chen et al., 2019). The findings revealed the presence of eight common types of barnyard grass in Chinese rice fields, namely *E. crus-galli* var. *Mitis*, *E. crus-galli* var. *crus-galli*, *E. crus-galli* var. *Zelayensis*, *E. oryzoides*, *E. cruspavonis*, *E. colona*, and *E. caudata*. Among the eight common types of barnyard grass, *E. glabrescens* ranked second with an occurrence frequency of 47.28% (Chen et al., 2019).

Currently, herbicide application remains a pivotal approach for weed management in China. To enhance rice production, it is essential to use herbicides to control barnyard grass in rice fields. However, the emergence of barnyard grass resistance owing to prolonged herbicide use presents a significant challenge for weed management in rice fields (Butts et al., 2022). Therefore, investigating the underlying mechanisms of barnyard grass resistance to herbicides can enhance weed management in rice fields, facilitate a more informed selection of herbicides, and reduce their usage, thereby contributing to the conservation of the ecological environment. Recently, research on *Echinocloa* spp. has primarily focused on *E. crus-galli*, with comparatively less investigation conducted on *E. glabrescens* as a subject of study. However, Chinese researchers have reported a higher frequency of *E. glabrescens* in Chinese paddy fields than in *E. crus-galli* (Chen et al., 2019). Therefore, it is imperative to use *E. glabrescens* as an experimental specimen in extensive research.

Florpyrauxifen-benzyl (FPB), a synthetic auxin herbicide (SAH) developed by Dow AgroSciences, Inc., was registered in China in 2017 and has been used as a foliar herbicide to control barnyard grass in paddy fields (Jin et al., 2023). Owing to its remarkable efficacy against rice weeds and non-toxicity to rice plants, it has gained widespread use in rice cultivation (Wright et al., 2020; Velásquez et al., 2021). Additionally, FPB is being utilized for the management of weeds that have developed resistance to conventional herbicides owing to its distinct target site (Miller et al., 2018).

The mode of action of FPB is analogous to that of other synthetic auxin herbicides, mimicking the excessive levels of the natural plant hormone indole-3-acetic acid (IAA). This disruption affects endogenous plant hormone balance, significantly affecting plant growth and development, interfering with nutrient transport, and ultimately leading to plant mortality (Grossmann, 2000). However, because of the intricate cascade of reactions they induce within plants, the precise mechanism by which SAHs exert their weed-killing effects remains elusive. Furthermore, it has been demonstrated that the receptor involved in auxin signaling for FPB exhibits distinct characteristics compared with older SAHs such as quinclorac and 2,4-dichlorophenoxyacetic acid (Walsh et al., 2006). Specifically, FPB shows a higher affinity for AFB5 than for TIR1 (which is commonly known as the receptor for quinclorac and 2,4-dichlorophenoxyacetic acid) (Lee et al., 2014; Epp et al., 2016). Consequently, FPB can effectively control barnyard grass populations that have developed quinclorac resistance (Miller et al., 2018).

Several reports have documented barnyard grass resistance to FPB, with one study conducting greenhouse screening of multiple barnyard grass populations in the south-central U.S., identifying populations that exhibited resistance to FPB. A subsequent investigation revealed that resistant barnyard grass showed lower FPB uptake efficiencies than their susceptible counterparts, suggesting that this is a potential mechanism underlying the development of resistance (Hwang et al., 2022a, b). In a recent study conducted by our research team, differences in phytohormone signaling between resistant and susceptible barnyard grasses were compared after treatment with FPB, using transcriptomic technology. The findings revealed that susceptible barnyard grasses showed increased expression of genes associated with ethylene, abscisic acid, and brassinolide synthesis and signaling after FPB treatment (Jin et al., 2023). Resistance to both FPB and penoxsulam in a barnyard grass population was observed in a recent investigation, suggesting the presence of pre-existing P450-mediated resistance to these herbicides before the commercialization of FPB (Takano et al., 2023). Meanwhile, Wang et al. (2024) discovered that *EcCYP72A385* was regulated by *EcMETTL7A* in *Echinochloa crus-galli* (L.) P. Beauv, ultimately leading to resistance to FPB, which undoubtedly deepens our understanding of FPB metabolic resistance.

Progress in systems biology research has facilitated the application of advanced omics technologies such as transcriptomics and metabolomics, enabling a comprehensive analysis of the underlying mechanisms of herbicide toxicity and weed resistance to herbicides (Küpper et al., 2018; Lygin et al., 2018; Concepcion et al., 2021). Transcriptomic techniques have been extensively used to identify weed herbicide resistance genes, particularly those associated with non-target resistance (Zhao et al., 2017; He et al., 2023; Takano et al., 2023). In a recent study, transcriptome differences between glufosinate-resistant *Eleusine indica* and glufosinate-susceptible *Eleusine indica* were compared, revealing a GST gene and three P450 genes exhibiting higher expression levels in the transcriptome of glufosinate-resistant *Eleusine indica*, suggesting that these genes confer resistance to glufosinate (He et al., 2023). In a recent comparative transcriptomic analysis between tripyrasulfone-resistant *Leptochloa chinensis* (L.) Nees and tripyrasulfone-susceptible *Leptochloa chinensis* (L.) Nees, researchers identified five P450 genes and a GST gene that potentially contribute to metabolic resistance to tripyrasulfone (Ju et al., 2023). While the application of metabolomic technology in weed resistance research is currently at the stage of experimental investigation and exploration, pioneering researchers have utilized Liquid Chromatograph Mass Spectrometer technology to investigate intermediate products and crucial metabolic pathways involved in herbicide metabolism (Concepcion et al., 2021; Strom et al., 2021; Concepcion et al., 2024). Concepcion et al. (2021) identified syncarpic acid-3 (SA3) metabolites via LC-MS-based untargeted metabolomics, revealed a novel reduction–dehydration–GSH conjugation detoxification mechanism. Concepcion et al. (2024) detected and characterized mesotrione metabolites in Palmer amaranth and proposed a detoxification pathway for mesotrione in Multiple-Herbicide-Resistant Palmer amaranth. Recently, an integrative study combining transcriptomic and metabolomic analyses in *Setaria italica* revealed that atrazine-resistant *Setaria italic*a may adapt to atrazine stress through glutathione synthesis and proline accumulation. These previous studies have convincingly demonstrated the efficacy of transcriptomic and metabolomic techniques as valuable tools for investigating herbicide resistance (Sun et al., 2022).

In the present study, we performed a comparative analysis of the transcript and metabolic profiles of an FPB-resistant *E. glabrescens* population and a susceptible *E. glabrescens* population after treatment with the recommended field dose of FPB, thereby providing novel insights into the underlying mechanisms of *E. glabrescens* resistance to FPB.

## 2 Materials and methods

### 2.1 Plant material and growth conditions

The two *E. glabrescens* populations utilized in this study were sourced from the barnyard grass germplasm repository of the Weed Research Group at China National Rice Research Institute (CNRRI, Hangzhou, China). The resistant *E. glabrescens* population was originally collected in Yuanjiang County, Hunan Province in 2017, while the susceptible *E. glabrescens* population was obtained from Yangzhou City, Jiangsu Province in 2018. The two *E. glabrescens* seeds utilized in this study were both G2 generations that had been purified from the originally collected seeds, and they exhibited stable FPB-resistant/-susceptible phenotypes.

In this study, the susceptible *E. glabrescens* population was utilized as a control group for conducting dose-response curve experiments, non-targeted metabolomics, and transcriptomics analyses. The cultivation process of the plant materials was conducted as follows: Initially, a piece of thick filter paper was placed flat in a transparent plastic petri dish with a diameter of 9 cm. Approximately 3 to 4 ml of water was then added to moisten the filter paper. Subsequently, about 150 to 200 *E. glabrescens* seeds (per petri dish) were evenly dispersed onto the moistened filter paper (resistant and susceptible *E. glabrescens* seeds were treated separately). The prepared dishes were subsequently placed inside a light-temperature incubator within the laboratory. The light-temperature conditions were set at alternating intervals of 12 hours darkness (20 °C) and 12 hours light (30 °C). To ensure continuous moisture for both the filter paper and sclerotium barnyard seeds, additional water was added into each Petri dish every 24 hours. In the second step, wait until the *E. glabrescens* seed germination occurs, and then delicately extract the germinated seeds using tweezers for transplantation into plastic small pots measuring 7.5cm in diameter and 7cm in height. The small pots are filled with soil to a depth of approximately 6cm, utilizing herbicide-free and high-temperature sterilized soil that has been adequately pre-moistened for optimal water retention. Germinated seeds are then planted at a depth of around 1cm. In the third step, the *E. glabrescens* seedlings were cultured in an artificial climate chamber at CNRRI, under identical growth conditions for both resistant and susceptible *E. glabrescens*. The temperature inside the chamber was maintained at 30°C during daytime (12 hours) and 20°C during nighttime (12 hours). White LED lights were utilized to simulate solar illumination, with a light intensity of 500 µmoles/m²/sec. Upon reaching the 2–3 leaf stage, *E. glabrescens* seedlings of comparable growth size were transplanted into small pots, with three seedlings per pot. The seedlings were regularly irrigated throughout their growth period to maintain soil moist while preventing waterlogging. Once *E. glabrescens* reached the 3–4 leaf stage, the subsequent phase of the experiment was initiated.

### 2.2 Dose–response experiments of FPB

The whole-plant bioassays were used to calculate the dose-response curves to obtain the herbicide dose required for 50% growth reduction (GR_50_) of the resistant/susceptible *E. glabrescens*. The leaf age of *E. glabrescens* used was 3–4 leaf stage, and the methods and instruments used were consistent with those used in our previous studies, with minor modifications (Jin et al., 2023). The specific distinction lies in the alteration of the FPB dose used, with a consistent application of FPB doses at 0, 1.13, 2.25, 4.50, 9.00, 18.00, 36.00 and 72.00 g a.i. ha^−1^ for the resistant/susceptible *E. glabrescens* in this experiment. After 14 days, the aboveground fresh weight was measured and used to calculate the relative fresh weight (the ratio of the treatment group to the control group). Non-linear regression analysis was conducted using the four-parameter Logistic regression model in OriginPro 2023 software to fit the dose-response curve and determine the GR_50_ value based on the relative fresh weight data and corresponding herbicide doses (He et al., 2023). The GR_50_ value is determined by the following formula:


$$\text{y}=\text{C}+\left(\text{D}-\text{C}\right)/\left[1+{\left(\text{x}/\text{G}{\text{R}}_{50}\right)}^{\text{b}}\right]$$

Where y refers to the relative fresh weight corresponding to a specific treatment, D refers to the maximum dose response value, C refers to the minimum dose response value, and b refers to the slope at the GR_50_ value. The ratio of the GR_50_ values for resistant *E. glabrescens* and susceptible *E. glabrescens* was designated as the resistance index (RI), serving to indicate the level of resistance in the resistant *E. glabrescens*.

### 2.3 FPB treatments and sample preparation for untargeted metabolomics and transcriptomics sequencing

The FPB treatments were applied to *E. glabrescens* at the 3–4 leaf stage using a laboratory spray tower, which was equipped with information and parameters consistent with those used in our previous studies. The FPB treatments were applied at a dose of 36 g a.i. ha^-1^, which represents the maximum recommended dose for field application.

Based on our observation of the growth status of *E. glabrescens* following FPB treatment, susceptible *E. glabrescens* exhibited evident symptoms of herbicide exposure approximately 72 hours after treatment. Considering that molecular changes leading to phenotypic alterations occur earlier, we chose to analyze the old leaves of resistant/susceptible *E. glabrescens* at 48 h after treatment for conducting metabolomic analysis and transcriptome sequencing.

The plant material used for metabolomics analysis consisted of 6 biological replicates, totaling 12 samples from resistant/susceptible *E. glabrescens*; whereas the material utilized for transcriptome sequencing comprised 3 biological replicates, totaling 6 samples from resistant/susceptible *E. glabrescens*. The resistant *E. glabrescens* samples were designated as “YFR”, while the susceptible *E. glabrescens* samples were labeled as “YFS”, with approximately 1g of old leaves collected from each sample. The cut leaves were promptly enveloped in aluminum foil and subjected to rapid freezing by immersion in liquid nitrogen. Following the completion of sample collection, all samples were retrieved from liquid nitrogen and preserved at -80°C for subsequent untargeted metabolomics analysis, transcriptome sequencing, and RT-qPCR validation.

### 2.4 Metabolite extraction and detection

A total of 12 samples of YFR and YFS were used for metabolome analysis (6 biological replicates per group). The extraction of metabolites from each sample followed the subsequent procedures: Step 1: The sample stored at -80 °C in the refrigerator was pulverized into powder using liquid nitrogen; Step 2: 100 mg of the powdered material was transferred into an EP tube, followed by the addition of 500 μL of an aqueous solution containing 80% methanol. The mixture was thoroughly vortexed to ensure proper mixing; Step 3: After a brief immersion in an ice bath for 5 minutes, the sample underwent centrifugation at a temperature of 4 °C and a force of 15000×g for a duration of 20 minutes; Step 4: The supernatant was extracted and diluted with water graded for mass spectrometry to achieve approximately a methanol content of 53%; Step5: Another round of centrifugation under identical conditions (i.e., temperature =4°C, force=15000×g) was performedfor another duration of 20 min. Collect the resulting supernatantand storeitina vialfor subsequent LC-MS analysis. The extraction, qualification, and quantification of metabolites were conducted by Beijing Novogene Technology Co., Ltd.

The extraction of metabolites and the subsequent qualitative and quantitative analysis were carried out on YFR and YFS following a standardized protocol conducted by Beijing Novogene Technology Co., Ltd, with reference to established methods and procedures for metabolite extraction from tissue samples (Want et al., 2013). Considering the limited number of metabolomics studies on weed resistance and our objective to obtain a more comprehensive metabolite profile in YFR/YFS, we have opted for the untargeted metabolomics approach in this study.

Untargeted metabolomics technology is capable of comprehensively detecting molecular characteristic peaks in the samples, and then combining with the standard libraries constructed from high-quality mzCloud databases, mzVault and MassList databases to realize the matching identification of molecular characteristic peaks, so as to find out as much as possible the metabolites in the biological system and comprehensively reflect the information of total metabolites. The UHPLC-MS/MS analyses were performed at Novogene Co., Ltd. (Beijing, China) utilizing a Vanquish UHPLC system (ThermoFisher, Germany) connected to an Orbitrap Q Exactive TM HF mass spectrometer (Thermo Fisher, Germany). Equal volume samples were taken from each experimental sample and mixed as Quality Control (QC) samples (3 repetitions) to detect whether the stability and signal response strength of the instrument were normal (Rao et al., 2016). The specific quality control process involved the initial 3 QC samples before sample injection, which were utilized to monitor the instrument status and ensure equilibrium of the chromatography-mass spectrometry system. Subsequently, the following 3 QC samples were used for segmented scanning in conjunction with the secondary chromatogram obtained from the experimental sample to facilitate metabolite identification. QC samples inserted in the sample detection process served to assess system stability throughout the experiment and conduct data quality control analysis (Concepcion et al., 2021; Strom et al., 2021).

### 2.5 Metabolite annotation and analysis

The KEGG database (https://www.genome.jp/kegg/pathway.html), HMDB database (https://hmdb.ca/metabolites), and LIPIDMaps database (http://www.lipidmaps.org/) were used to metabolite annotation. For multivariate analysis, we utilized metaX software to perform data transformation and subsequently conducted principal component analysis (PCA) and orthogonal partial least squares discrimination analysis (OPLS-DA) to classify distinct groups and determine the variable importance in the projection (VIP) associated with each metabolite (Westerhuis et al., 2010; Wen et al., 2017). In univariate analysis, a t-test was used to determine the statistical significance (P-value) of each metabolite between YFR and YFS, while fold change (FC) of each metabolite between YFR and YFS was calculated. The conditions for screening differential metabolites included VIP > 1.0, FC > 1.5 or FC< 0.667, and P-value< 0.05 (Sreekumar et al., 2009; Haspel et al., 2014). Volcano plots were generated using the R package ggplot2 to visualize the overall distribution of these differential metabolites. The data were normalized by calculating z-scores based on the intensity areas of the differential metabolites before data analysis and visualization, and clustering heat maps were created using the Pheatmap package in R language (Wu et al., 2021).

### 2.6 Transcriptome sequencing and data analysis

A total of 6 samples of resistant and susceptible *E. glabrescens* were used for transcriptome sequencing (3 biological replicates per group). The total RNA extraction, cDNA library construction and purification, as well as transcriptome sequencing were performed by Beijing Novogene Technology Co., Ltd. Total RNA was extracted from the samples using the RNeasy Plant Mini Kit (QIAGEN, Germany) according to the manufacturer’s instructions provided with the kit. Novogene used the RNeasy Plant Mini Kit (QIAGEN, Germany) for total RNA extraction, following the manufacturer’s instructions. The purity of total RNA was assessed using a NanoPhotometer^®^ spectrophotometer, while the quantity and integrity were evaluated using an Agilent Bioanalyzer 2100 system (Agilent Technologies, USA).

PolyA-tailed mRNA was extracted from total RNA using Oligo(dT) magnetic beads, and subsequently subjected to random fragmentation to obtain fragmented mRNA templates for cDNA synthesis. After the synthesis of double-stranded cDNA, AMPure XP beads were used for cDNA purification and screening of fragments ranging from approximately 370 to 420 bp in length for subsequent PCR amplification. Subsequently, a second round of AMPure XP beads purification was performed on the PCR products, ultimately completing library construction. The cDNA library underwent quantitative and qualitative assessment using the Qubit 2.0 Fluorometer and Agilent 2100 bioanalyzer. Once the library was qualified, sequencing was performed on the Illumina NovaSeq 6000 platform.

The raw data underwent a series of preprocessing steps to ensure the generation of high-quality clean reads for downstream analyses. This included the removal of adapter-containing reads, reads containing N base (N refer to the base information cannot be determined), and low-quality reads (the proportion of reads with Qphred ≤ 20 bases exceeding 50% of the entire read length). The reference genome utilized in this study was obtained from the China National Center for Bioinformation (CNCB) and corresponds to *Echinochloa crus-galli* (Wu et al., 2022). The reference genome index was constructed by using the software HISA T2 (v2.0.5), and then the clean reads were compared with the reference genome of *Echinochloa crus-galli* to obtain the location information of reads on the reference genome and the characteristic sequence information of the sequenced samples (Mortazavi et al., 2008). The featureCounts tool in subread software was used to count the number of reads covered by each gene from the beginning to the end according to the location information of gene alignment on the reference genome (Liao et al., 2014). The reads with alignment mass value less than 10, reads on non-conforming alignment and reads alignment to multiple regions of the genome were filtered out. The transcript expression levels were quantified by FPKM values, which represent the expected number of Fragments Per Kilobase of transcript sequence per Millions base pairs sequenced (Bray et al., 2016).

The R packages corrplot and ggplot2 were utilized to compute the intra- and inter-group correlation coefficients based on the FPKM values of all genes in each sample, followed by generating an inter-sample correlation heatmap. The DESeq2 R package was utilized to perform differential expression analysis between sample groups, enabling the identification of a set of genes that exhibited differential expression across different sample groups (Love et al., 2014). The False Discovery Rate (FDR) value, commonly represented as adjusted p-values (padj), was obtained by applying the Benjamini-Hochberg (BH) method to adjust P-values for multiple-hypothesis testing (Anders and Huber, 2010; Young et al., 2010). The screening criteria for differentially expressed genes (DEGs) were defined as |log2(FoldChange)| ≥ 1 and a padj value ≤ 0.05 (Yu et al., 2020; Jin et al., 2023). After screening the DEGs, we utilized the R package ggplot2 to generate a volcano plot illustrating the overall distribution of these genes. Subsequently, we performed gene ontology (GO) function enrichment analysis and KEGG pathway enrichment analysis on the set of differential genes using the R package clusterProfiler (Wu et al., 2021).

### 2.7 Validation of RNA-seq by RT-qPCR

To validate the reliability of RNA-seq data, real time quantitative pcr (RT-qPCR) was used to ascertain whether the differential expression patterns of DEGs between YFR and YFS were consistent with the transcriptome findings. The kit and experimental procedure used in RT-qPCR were consistent with those used in our previous studies (Yu et al., 2020; Jin et al., 2023). We randomly selected 10 differentially expressed genes and 3 GST genes (BH02.4558, AH02.2825, BH02.1736) from the differentially expressed genes in both YFR and YFS, and a total of 13 DEGs were selected for RT-qPCR. We used the coding sequence of the selected gene to design the specific primers of RT-qPCR, and used actin as the housekeeping gene. The primer sequences were designed using Primer 5 software, and all primer sequences are provided in **Supplementary Table 1**. The quantification of each gene in each sample was repeated for 4 times, and the average value of the four technical repeats was taken to calculate the relative expression level using the 2^−ΔΔCt^ method. Subsequently, the log2FoldChange between YFR and YFS of the selected gene was calculated according to the relative expression level obtained from RT-qPCR results, and the correlation analysis was conducted with the log2FoldChange between YFR and YFS of the gene obtained from RNA-seq data.

### 2.8 Detection of GST-mediated resistance

4-chloro-7-nitrobenzoxadiazole (NBD-Cl) is a glutathione S-transferase (GST) suicide inhibitor widely used to detect the presence of GST-mediated resistance in weeds; if the resistant plants had demonstrated increased sensitivity to herbicides following treatment with NBD-Cl, it could have been inferred that the observed resistance was attributed to GST-mediated herbicide resistance (Ricci et al., 2005; He et al., 2023; Ju et al., 2023). We used the same spray tower used in FPB treatment to pretreat YFR with NBD-Cl. The treatment dose of NBD-Cl was 270 g a.i. ha^-1^, which is a recommended dose that is recognized to reduce GST activity without affecting normal plant growth (He et al., 2023; Ju et al., 2023). NBD-Cl pretreatment was performed 48h before FPB treatment. In this experiment, three doses of FPB treatment were applied, respectively 36 g a.i. ha-1, 18 g a.i. ha^-1^, and 9 g a.i. ha^-1^. After 14 days of FPB treatment, the fresh weight of the aboveground portion (aboveground biomass) of YFR plants was measured to assess the resistance level against FPB.

## 3 Results

### 3.1 FPB efficacy against resistant and susceptible *E. glabrescens*

The dose–response curves indicated that the resistant *E. glabrescens* exhibited resistance to FPB, with a resistance index (RI) of 10.65 (**Supplementary Figure 1**). Furthermore, the GR_50_ value of the susceptible *E. glabrescens* was 1.62 g a.i. ha^−1^, and the GR_50_ value of resistant *E. glabrescens* was 17.26 g a.i. ha^−1^. When the FPB dose was 18 g a.i. ha^−1^, the relative fresh weight of susceptible *E. glabrescens* plants had decreased to less than 10% after 14 days of FPB–treatment, while the relative fresh weight of resistant *E. glabrescens* plants remained above 50%. At a FPB dose of 36 g a.i. ha^−1^, the aboveground parts of susceptible *E. glabrescens* plants had completely withered after 14 days of FPB–treatment, approaching zero relative fresh weight, whereas that of resistant *E. glabrescens* was still above 20%. The field-recommended maximum dose for FPB is 36 g a.i. ha^−1^; however, even at this level, it fails to achieve over 90% control in terms of fresh weight for resistant *E. glabrescens* under laboratory conditions.

### 3.2 Differential metabolite accumulation between YFR and YFS

In this study, a untargeted metabolomics detection approach based on UPLC-MS/MS technology was applied to qualitatively and quantitatively analyze metabolites in YFR and YFS. Based on a comprehensive search of mzCloud, mzVault, and MassList databases, a total of 1,243 metabolites were successfully identified in both YFR and YFS. The top five predominant metabolite classifications observed were as follows: Lipids and lipid-like molecules (31.89%), Organic acids and derivatives (14.55%), Phenylpropanoids and polyketides (14.24%), Organoheterocyclic compounds (13.21%), and Benzenoids (8.67%) (**Supplementary Figure 2**).

**Figure 1:**
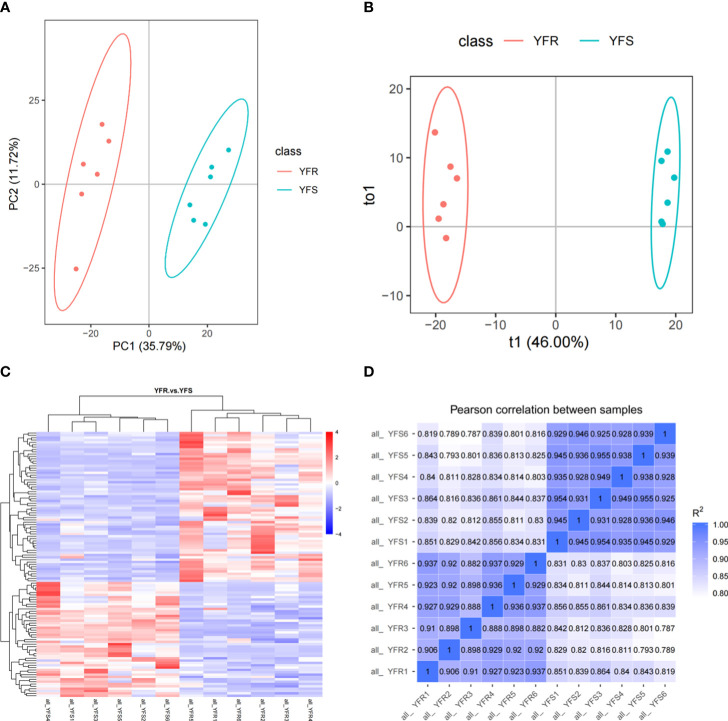
Differences in overall metabolic profile between YFR and YFS. **(A)** The PCA plot of all metabolome samples (YFR and YFS). **(B)** The PLS-DA plot of all metabolome samples. **(C)** The heat map of hierarchical cluster analysis of all metabolome samples. **(D)** The correlation heat map of all metabolome samples.

The Pearson correlation coefficient between QC samples was calculated based on the relative quantitative value of metabolites (**Supplementary Figure 3**). The results showed that the R^2^ values between different QC samples were all greater than 0.99, indicating that the instrument was stable and the data quality was reliable during the whole detection process. The PCA score and cluster analysis heat map demonstrated robust intra-group biological repeatability, while revealing significant disparities in metabolic profiles between YFR and YFS (**Figure 1A, C**). Meanwhile, the correlation analysis among samples demonstrated robust intra-group repeatability and discernible inter-group distinctions (**Figure 1D**).

After performing principal component analysis (PCA) and orthogonal partial least squares discriminant analysis (OPLS-DA), we conducted a difference analysis to compare the sample grouping (YFR vs YFS). The results obtained from both PCA and OPLS-DA consistently demonstrate a significant separation between the sample groups, while also highlighting the excellent repeatability of samples within each group (**Figure 1A, B**). In addition, in the ranking validation plot of the OPLS-DA model, as the horizontal coordinate replacement retention decreases, both R^2^ and Q^2^ decrease, and the regression line shows an upward trend, which indicates that the model is constructed accurately and there is no “overfitting” phenomenon (**Supplementary Figure 4**). The variable projection importance (VIP) value of the first principal component obtained from the OPLS-DA results is a crucial parameter for differential metabolite screening, as it quantifies the contribution of metabolites to group separation. In addition, the fold change (FC), obtained by calculating the ratio of the mean quantitative values across all biological replicates for each metabolite in the comparison group, and the P-value, which signifies the level of significance for observed differences and is determined through T-test analysis, serve as two additional parameters utilized to screen differential metabolite references.

**Figure 2:**
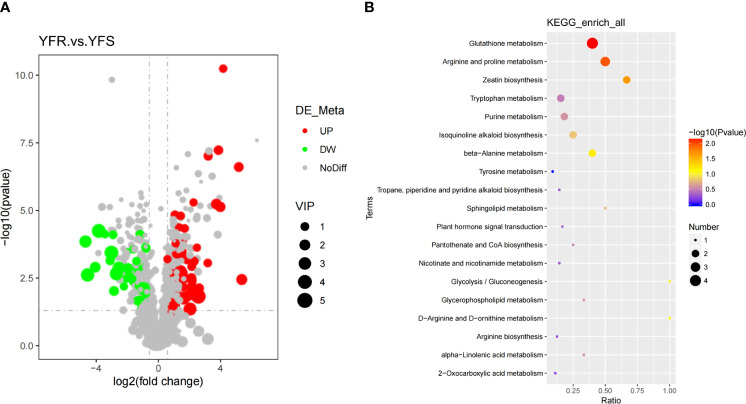
Analysis of Differential Metabolites. **(A)** The volcano plot of DAMs between YFR and YFS. **(B)** The KEGG enrichment bubble plot of DAMs between YFR and YFS.

In this study, we identified metabolites that satisfied the criteria of VIP > 1.0, FC > 1.5 or FC< 0.667 with a P-value< 0.05 as significantly different between the comparison groups. The clustering heatmap of DAMs was clearly illustrated the discernible metabolic distinctions between YFR and YFS, while the volcano plot visually depicted the overall distribution pattern of these differentially metabolites (**Figures 1C**, **2A**). In total, 1243 metabolites were detected in both YFR and YFS, with 115 DAMs exhibiting significant differences (65 up-regulated and 50 down-regulated). We hypothesized that the differential accumulation of these 115 metabolites may be associated with the difference in tolerance to FPB between YFR and YFS (**Supplementary Table 2**). The enrichment of DAMs in different pathways in the comparison combination between YFR and YFS was demonstrated by the KEGG enrichment bubble plot (**Figure 2B**). These differential metabolites were significantly enriched in Glutathione metabolism (p-value=0.00720), Arginine and proline metabolism (p-value=0.0106), and Zeatin biosynthesis (p-value=0.0221) pathways (**Supplementary Table 3**).

**Figure 3:**
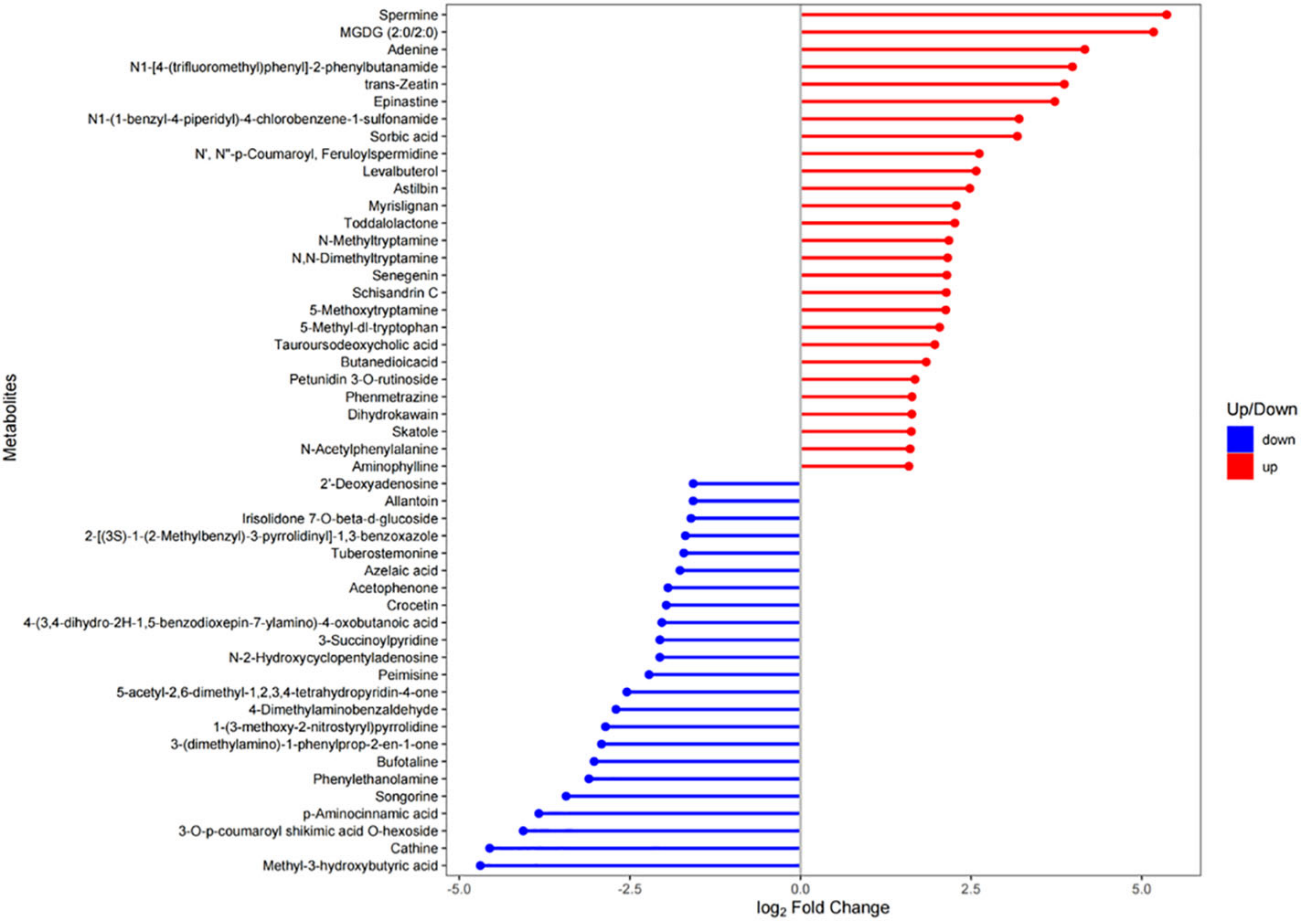
Matchstick plot of the top50 DAMs between YFR and YFS.

The matchstick plot was used to illustrate the differential expression of |log2FC| in the top 50 DAMs, with 27 being upregulated and 23 downregulated (**Figure 3**). Among the upregulated metabolites, Spermine, MGDG (2:0/2:0), Adenine, N1-[4-(trifluoromethyl)phenyl]-2-phenylbutanamide, and trans-Zeatin exhibited the highest |log2FC| values (top 5). Conversely, Methyl-3-hydroxybutyric acid, Cathine, 3-O-p-coumaroyl shikimic acid O-hexoside, p-Aminocinnamic acid and Songorine exhibited the highest |log2FC| values (top 5) in expression among the downregulated metabolites. Overall, Spermine (upregulated), MGDG (2:0/2:0) (upregulated), Methyl-3-hydroxybutyric acid (downregulated), Cathine (downregulated), and Adenine (upregulated) were identified as the top five metabolites based on their |log2FC| values (**Supplementary Table 4**). Interestingly, spermine, which exhibited the highest |log2FC| value, was found to be associated with two significantly enriched pathways: Glutathione metabolism and Arginine and proline metabolism. Moreover, adenine and trans-zeatin, ranking fifth and eighth in terms of |log2FC| values respectively, were linked to Zeatin biosynthesis pathway. However, other metabolites displaying high ranks in |log2FC| values did not exhibit associations with any significantly enriched pathways.

### 3.3 Differential gene expression between YFR and YFS

**Figure 4:**
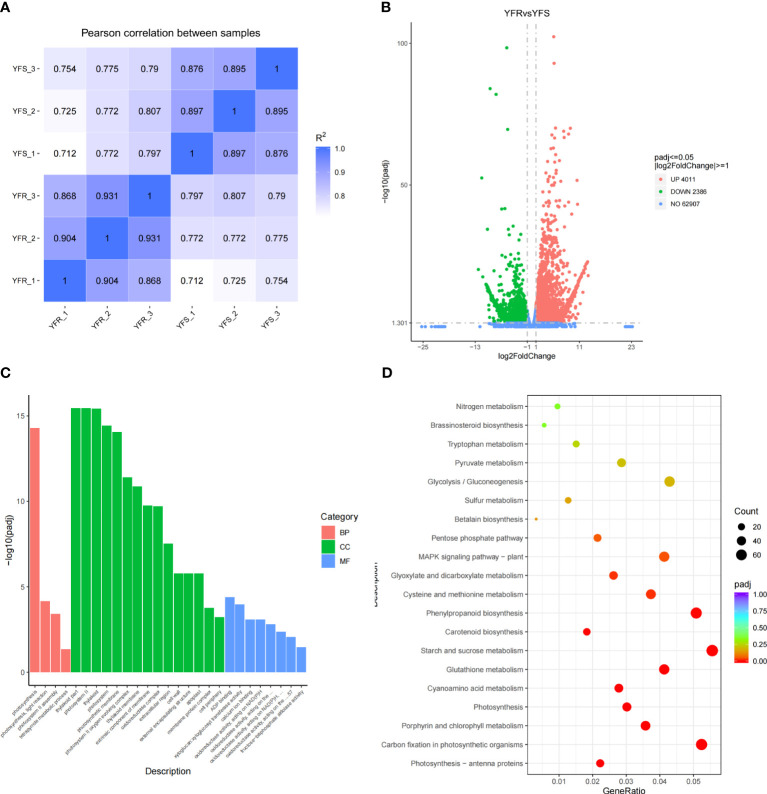
Differential gene expression between YFR and YFS. **(A)** The correlation heat map of all transcriptome samples (YFR and YFS). **(B)** The volcano plot of DEGs between YFR and YFS. **(C)** The histogram of GO significant enrichment of DEG between YFR and YFS. **(D)** The KEGG enrichment bubble plot of DEGs between YFR and YFS.

The transcriptomes of YFR and YFS were sequenced using RNA-seq technology on the Illumina NovaSeq 6000 sequencing platform in this study. A total of 279,176,734 raw reads were obtained from 6 samples. After undergoing quality control processing, a final count of 272,598,074 clean reads was ultimately obtained. The Clean reads of each sample were assessed for Q20, Q30, and GC contents. It was observed that the Q20 base percentages of Clean reads in all analyzed samples exceeded 95%, while the Q30 base percentages surpassed 88% (**Supplementary Table 5**). Additionally, the GC contents ranged between 56% and 59%. The clean reads of each sample were aligned to the reference genome of *Echinochloa crus-galli*, obtained from the CNCB (Wu et al., 2022). The alignment success rate in the reference genome of *Echinochloa crus-galli* exceeded 87%, with a unique mapping rate exceeding 83% (**Supplementary Table 5**). The high degree of alignment efficiency observed between the transcriptome data and reference genome suggests the accuracy of the selected reference genome and transcriptome sequencing results. It was suggested that the chosen reference genome assembly met the requirements for information analysis. The correlation analysis among samples was conducted based on the FPKM values of all genes in each sample. The correlation heatmap revealed that the squared Pearson correlation coefficient (R^2^) exceeded 0.85 for all groups, indicating robust repeatability among samples within the same group (**Figure 4A**). Additionally, the intragroup samples exhibited a higher correlation compared to the intergroup samples, suggesting distinct gene expression patterns between YFR and YFS.

The R package DESeq2 was utilized for conducting differential expression analysis, and the identification of DEGs was performed based on the screening criteria of |log2(FoldChange)|≥1 and FDR<0.05. The volcano plot was presented as a visual depiction illustrating the overall distribution of DEGs (**Figure 4B**). In order to gain further insights into the genetic disparities between YFR and YFS, as well as identify potential biological pathways associated with the FPB-resistance mechanism of YFR, we performed KEGG and GO enrichment analyses on DEGs. The results of GO enrichment analysis revealed a significant enrichment of DEGs in 27 GO Terms, with 15 GO Terms classified under the cellular component category, 8 under the Molecular Function category, and 4 GO Terms under the biological process category (**Figure 4C**). Among these GO Terms, the top five with padj values were thylakoid part, photosystem II, thylakoid, photosynthesis, and photosystem. The results of KEGG enrichment analysis revealed a significant enrichment of DEGs in 12 KEGG pathway terms, including photosynthesis - antenna proteins, carbon fixation in photosynthetic organisms, porphyrin and chlorophyll metabolism, photosynthesis, cyanoamino acid metabolism, glutathione metabolism, starch and sucrose metabolism, carotenoid biosynthesis, phenylpropanoid biosynthesis, cysteine and methionine metabolism, glyoxylate and dicarboxylate metabolism and MAPK signaling pathway - plant (**Figure 4D**). According to the results of KEGG and GO enrichment analysis, significant differences in photosynthesis were observed between YFR and YFS. Additionally, it is noteworthy that the glutathione metabolism pathway emerged as a significantly enriched pathway for both differentially expressed genes and metabolites. This means that the glutathione metabolism pathway may play an important role in the resistance of YFR to FPB.

In the YFRvsYFS comparison combination, 4 DAMs were enriched into the Glutathione metabolism pathway, namely Spermine (Com_1942_pos), Cys-Gly (Com_9871_pos), L-Ornithine (Com_2045_neg) and Oxidized glutathione (Com_3100_neg). Among them, Spermine and L-Ornithine had higher relative contents in YFR, while Cys-Gly and Oxidized glutathione had higher relative contents in YFS; 52 DEGs were enriched into the Glutathione metabolism pathway, and these gene clusters were involved in the encoding of glutathione S-transferase (GST), peroxidase (POD), spermidine synthase (SPDS), isocitrate dehydrogenase (ICDHP), γ-glutamylcyclotransferase (GCT), glucose-6-phosphate 1-dehydrogenase (G6PD), and glutathione synthetase (GSH). Among them, the gene clusters encoding GST, SPDS, POD, ICDHP, and GCT had higher relative expression levels in YFR, while the gene clusters encoding GSH and G6PD had higher relative expression levels in YFS. Interestingly, L-Ornithine serves as a precursor in the spermine biosynthesis, while spermine synthase encoded by SPDS gene plays a pivotal role as an essential enzyme in this process. Consequently, the elevated relative content of spermine observed in YFR may be attributed to its enhanced synthesis. The overall findings indicated that the higher relative content of spermine in YFR and the elevated relative expression of genes encoding GST and POD in YFR may have both played a pivotal role in the resistance mechanism of YFR to FPB.

### 3.4 RT-qPCR validation of DEGs between YFR and YFS

**Figure 5:**
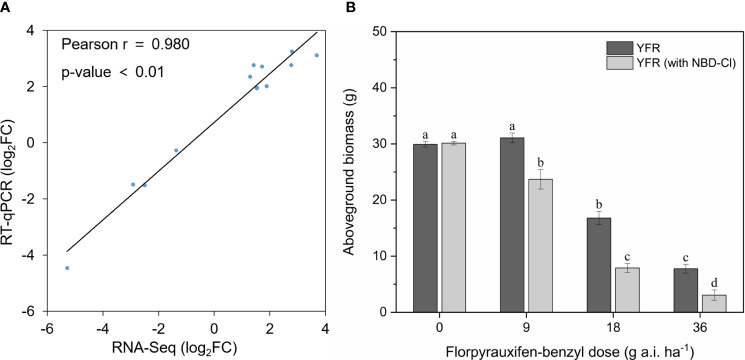
Revalidation of RNA-seq data through qPCR and verification of GST-mediated resistance via GST inhibitors. **(A)** The correlation between the RT-qPCR results and RNA-Seq data of selected genes. **(B)** The histograms of YFR’s aboveground biomass under four FPB doses with and without NBD-Cl pretreatment. Different lowercase letters above errors bar represent differences (P < 0.05) between treatments.

To validate the reliability of the transcriptome data, we used RT-qPCR to confirm the expression levels of 13 DEGs in YFR and YFS, respectively. 10 DEGs were randomly selected from the pool of all DEGs in both YFR and YFS, while the remaining 3 DEGs (GSTU8, GSTU18, GSTF1) were specifically associated with the glutathione metabolism pathway. The consistent trend in the results of RT-qPCR (log2FC) and RNA-seq (log2FC) was revealed by the correlation scatter diagram, indicating a high correlation between the two results (**Figure 5A**; **Supplementary Table 6**). It is suggested that the transcriptome data obtained in this study were reliable. Furthermore, the genes GSTU8, GSTU18, and GSTF1 involved in the glutathione metabolism pathway consistently demonstrate higher expression levels in YFR as revealed by RNA-seq data.

### 3.5 NBD-Cl Reduced FPB-Resistance in YFR

To ascertain the existence of GST-mediated resistance to FPB in YFR, we pre-treated YFR with the GST inhibitor NBD-Cl prior to FPB treatment and examined the impact of this intervention on FPB resistance. The results demonstrated that the growth of YFR remained unaffected by NBD-Cl pretreatment, while a decrease in FPB resistance of YFR was observed after NBD-Cl pretreatment. As depicted in **Figure 5B**, no significant disparity was observed in aboveground biomass between NBD-Cl pretreated YFR and untreated YFR after a 14-day growth period. However, following the same dose of FPB treatment, YFR pretreated with NBD-Cl exhibited a significantly lower aboveground biomass than those without NBD-Cl pretreatment after a 14-day growth period. The inhibition of NBD-Cl on GST activity led to a reduction in FPB resistance of YFR, providing experimental evidence supporting the involvement of GST in the development of FPB resistance in YFR.

## 4 Discussion

### 4.1 The expression level of the GST gene was significantly higher in YFR

Glutathione S-transferase (GST) is a ubiquitous class of enzymes involved in the defense mechanisms of organisms (Nianiou-Obeidat et al., 2017). In plants, GST has been demonstrated to play a crucial role in herbicide detoxification and metabolic processes (Dixon et al., 2002). GST can facilitate the conjugation of herbicide/herbicide primary metabolites with GSH within plant cells, leading to either diminished herbicidal activity or enhanced susceptibility to degradation by related metabolic enzymes, thereby enabling herbicide detoxification (Reinemer et al., 1996; Dixon et al., 2002). Since Shimabukuro’s initial discovery of GST-mediated atrazine-GSH binding in maize, which mitigates atrazine-induced crop damage, the pivotal role of GST in plant herbicide resistance has been extensively investigated (Shimabukuro et al., 1970). A plethora of studies have demonstrated the pivotal role of GST in the development of herbicide resistance in plants, encompassing classes such as sulfonylurea, chlorotriazine, chloroacetanilide, diphenyl ether, and aryloxy phenoxy propionate (Skipsey et al., 2005; Li et al., 2017; Nakka et al., 2017; Cao et al., 2023; Guan et al., 2023). Furthermore, herbicide-resistant plants typically exhibit elevated activity of GST enzymes or heightened expression levels of GST-encoding genes compared to their herbicide-sensitive counterparts (Li et al., 2023). The study conducted by Feng et al. (2022) revealed that the penoxsulam-resistant *Echinochloa crus-galli* population exhibited elevated GST enzyme activity and gene expression encoding GST (GST1–1, GST1–2, GST1–3) compared to the penoxsulam-sensitive population. The study conducted by Zhao et al. (2017) also revealed an upregulation of GST gene (GST-T3; GST-F1; GSTZ2; GSTT3) expression in mesosulfuron-methyl-resistant populations of Alopecurus aequalis, which aligns with the recent findings reported by Zhan et al. (2022).

In this study, we identified a higher expression level of 3 genes (GSTU8, GSTU18, GSTF1) encoding GST in YFR compared to YFS based on RNA-seq data and qPCR experiments. Furthermore, pretreatment with NBD-Cl enhanced the fresh weight control effect of FPB on YFR, indicating the presence of GST-mediated FPB resistance in YFR. Therefore, we postulated that GSTU8, GSTU18, and GSTF1 might have been involved in the resistance of YFR to FPB; however, further research was needed to elucidate the specific mechanism.

### 4.2 Spermine level in YFR was higher compared to that in YFS

Spermine (Spm) is a member of the polyamines (PAs), which have been widely recognized for their pivotal role in mediating diverse adaptive responses to environmental stresses and are considered as crucial molecules for plant stress adaptation (Gupta et al., 2013; Liu et al., 2015). Alterations in polyamine levels are frequently observed in plants under abiotic stresses, including drought, low temperature, and high salinity (Liu et al., 2007). In certain reports, the levels of the three most prevalent polyamines (putrescine, spermidine, and spermine) exhibited a significant increase following abiotic stress (Amini et al., 2021); however, it was more common for only one of these polyamines to show an elevation (Liu et al., 2015; Antoniou et al., 2021). For instance, putrescine demonstrated a substantial increase in apple callus subjected to stress conditions, while spermidine exhibited a notable rise in orange callus exposed to salt and cold stress (Liu et al., 2006). Additionally, salt stress resulted in a significant accumulation of spermine in 2-day-old etiolated sunflower seedlings (Tailor et al., 2019). These findings imply that the accumulation of polyamines may be influenced by various factors including intraspecies variations, tissue-specific detection sites, and types of stress encountered. Furthermore, they provide evidence supporting the involvement of polyamines in plant responses to abiotic stress. In comparative experiments investigating tolerant and sensitive genotypes of the same plant under identical stress conditions, it has been traditionally believed that tolerant biotypes exhibit higher polyamine accumulation (Hatmi et al., 2015). However, distinct patterns of polyamine accumulation have been observed among different genotypes of the same plant. For instance, salt-tolerant sorghum genotype accumulates higher levels of Spd and Spm under salt stress, whereas sensitive sorghum genotype exhibits increased Put accumulation (de Oliveira et al., 2020).

The contribution of different types of polyamines to abiotic stress adaptation in plants remains unclear; however, it is hypothesized that Spd and Spm, particularly Spm, possess a higher number of primary amino groups (−NH_2_) compared to Put (Liu et al., 2015). This characteristic may potentially enhance their effectiveness in facilitating plant adaptation to stress. Currently, the accumulation of spermine in plants has been demonstrated to enhance plant adaptation to salt stress, while exogenous application of spermine has also been shown to partially mitigate salt stress-induced damage in salt-sensitive plants (Yamaguchi et al., 2006; da Silva et al., 2022). Moreover, spermine is also implicated in the regulation of reactive oxygen species in plants, thereby contributing to the maintenance of biofilm stability under stressful conditions. The external application experiment of spermine has demonstrated its potential in alleviating membrane lipid peroxidation and H_2_O_2_ accumulation under abiotic stress conditions, while simultaneously enhancing the activity levels of antioxidant enzymes, including SOD, POD, CAT, and APX, to mitigate O_2_^-^ content (ElSayed et al., 2018; Xu et al., 2021; Li et al., 2022). Furthermore, spermine has been proposed in several studies to function as a signaling molecule and play a pivotal role in defense reaction (Mitsuya et al., 2009; Sagor et al., 2009). However, the precise role of spermine in plant response to herbicide stress remains largely elusive, and only a limited number of studies have demonstrated that exogenous application of spermine can enhance plant tolerance to paraquat (Pascual et al., 2023).

In this study, based on the results of untargeted metabolomics and transcriptomics, we observed a higher level of Spm accumulation in YFR compared to YFS, suggesting that the elevated Spm accumulation in YFR may be attributed to increased synthesis of Spm. We hypothesized that YFR may adapt to FPB stress by upregulating Spm synthesis, leading to enhanced activity of antioxidant enzymes such as GST and POD. Consequently, this augmentation in enzymatic defense systems improves the efficiency of reactive oxygen species scavenging generated by FPB treatment and mitigates membrane lipid peroxidation, ultimately conferring resistance against FPB.

## 5 Conclusion

In this study, we identified an FPB-resistant *E. glabrescens* population with a resistance index (RI) of 10.65 and provided the initial application of untargeted metabolomics and transcriptomics to study FPB resistance in *Echinochloa glabrescens*. We conducted a comparative analysis using untargeted metabolomics and transcriptomics to investigate the differences between an FPB-resistant *E. glabrescens* population and a susceptible *E. glabrescens* population after treatment with the recommended field dose of FPB. Our findings indicate that GST (GSTU8, GSTU18, GSTF1) may mediate the resistance to FPB in *E. glabrescens* and we demonstrated the presence of GST-mediated metabolic resistance in an FPB-resistant *E. glabrescens* population by using NBD-Cl. Furthermore, our study has revealed for the first time that elevated levels of spermine accumulation in FPB-resistant *E. glabrescens* may also contribute to the development of FPB resistance, providing novel insights into the mechanisms of FPB resistance. However, there are still many limitations in this study due to the limitations of experimental conditions. For instance, while we were able to detect the presence of GST-mediated metabolic resistance using NBD-Cl, we were unable to determine the structures of the potential GST-FPB metabolites. Additionally, our research was based on one resistant *E. glabrescens* population and one susceptible population, without conducting extensive screening and validation; therefore, the conclusions drawn may not have been universally applicable. Furthermore, functional validation of the identified candidate genes for resistance had not been performed, and the specific relationship and mechanism between spermine accumulation and herbicide resistance remained unresolved. We intended to collaborate with researchers in future studies to address these limitations, particularly regarding the last two issues.

## Data availability statement

The data presented in the study are deposited in the NCBI Sequence Read Archive (SRA) database, accession number PRJNA1081765.

## Author contributions

WJ: Formal analysis, Investigation, Validation, Writing – original draft, Writing – review & editing, Data curation, Software. KX: Data curation, Investigation, Software, Visualization, Writing – original draft, Writing – review & editing. WT: Funding acquisition, Resources, Writing – review & editing. YY: Project administration, Supervision, Writing – review & editing. JZ: Project administration, Supervision, Writing – review & editing. XY: Conceptualization, Funding acquisition, Writing – original draft, Writing – review & editing, Methodology. YL: Conceptualization, Funding acquisition, Methodology, Writing – original draft, Writing – review & editing, Resources.
