# Supplementary material for: Comparative metabolomics and transcriptomics provide new insights into florpyrauxifen-benzyl resistance in Echinochloa glabrescens
Source: Front Plant Sci. 2024 Jul 3;15:1392460. doi: 10.3389/fpls.2024.1392460 (PMC11253777; doi:10.3389/fpls.2024.1392460)
Supplement: Supplementary file 2 [file DataSheet_2.docx]

Supplementary Material

# Supplementary Figures and Tables

## Supplementary Figures


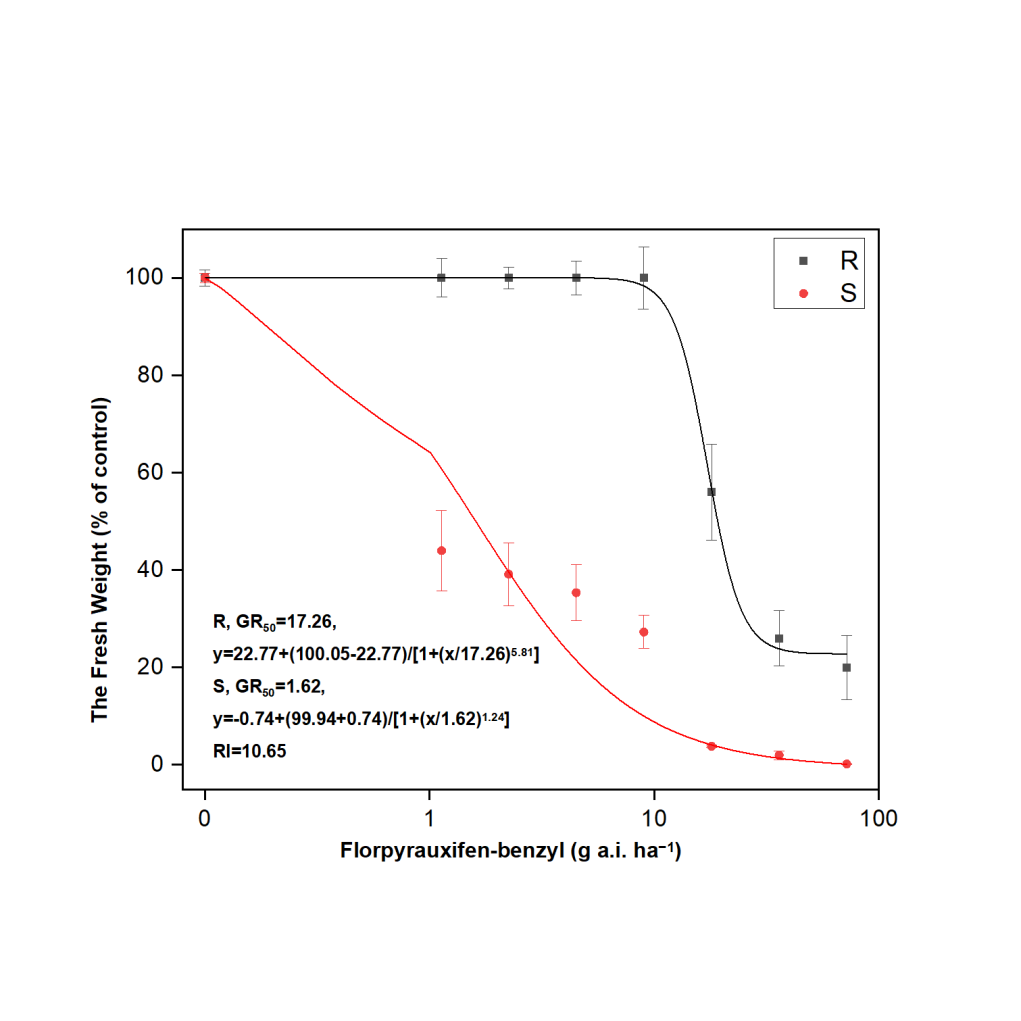


**Supplementary Figure 1.** Dose-response curves to FPB of resistant and susceptible *E. glabrescens*.


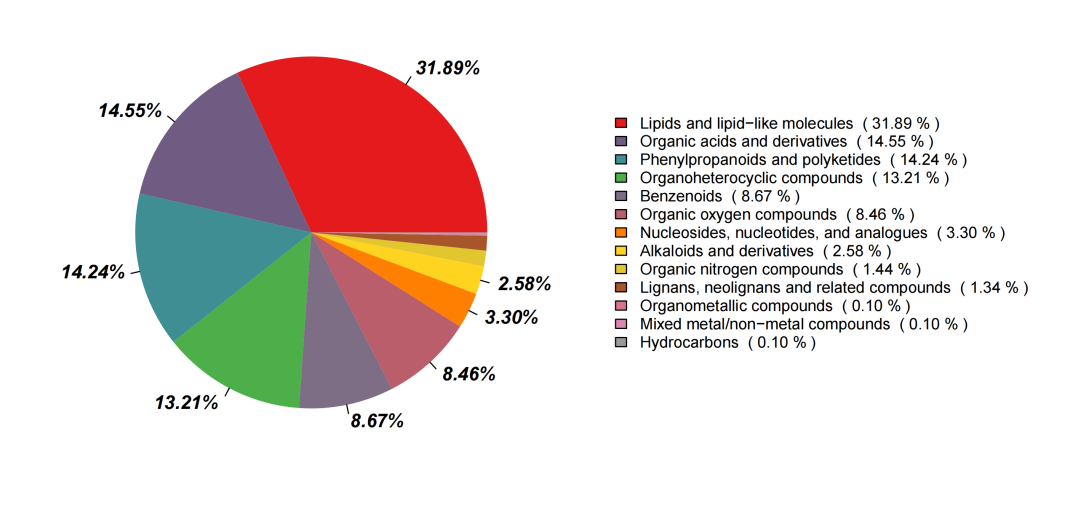


**Supplementary Figure 2.** The pie chart of all metabolite classification.


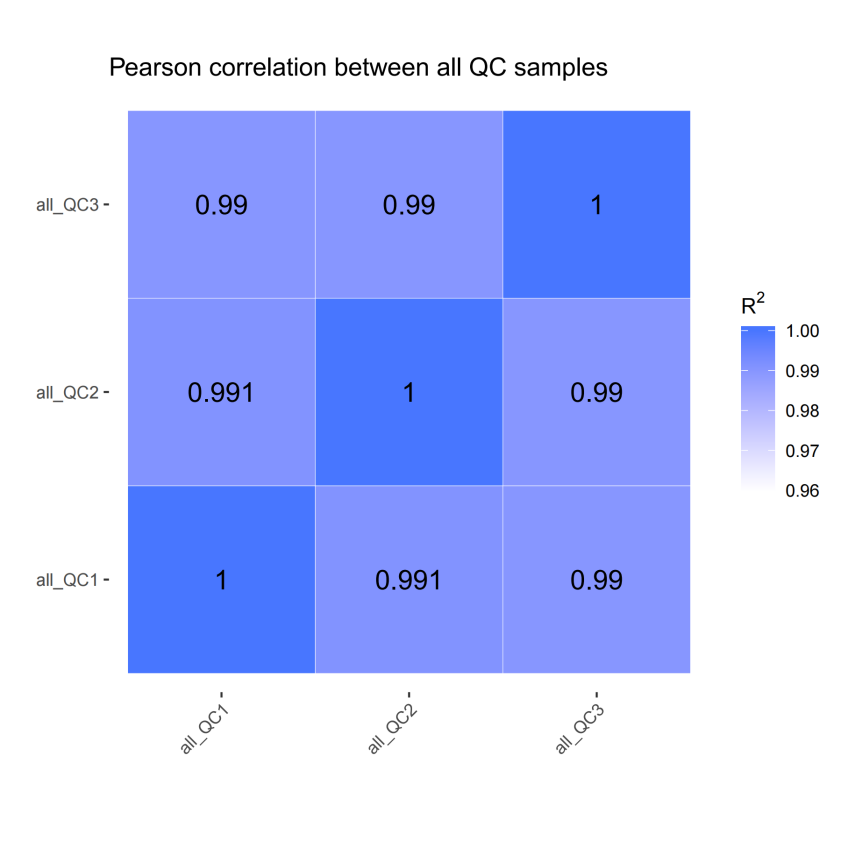


**Supplementary Figure 3.** The pearson correlation between all QC samples.


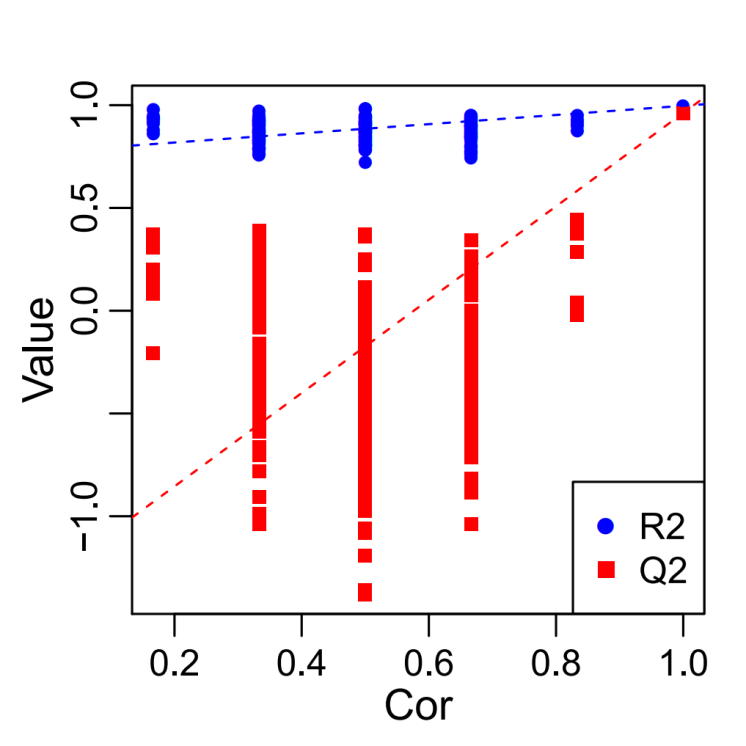


**Supplementary Figure 4.** The ranking validation plot of the OPLS-DA model.

## Supplementary Tables

There are 6 Supplementary Tables (Supplementary Table 1—6), which have been submitted as Excel.xlsx files in the Supplementary_Material.zip attachment.
